# Supplementary material for: Untargeted Metabolomic Analysis and Cytotoxicity of Extracts of the Marine Dinoflagellate Amphidinium eilatiense Against Human Cancer Cell Lines
Source: Toxins (Basel). 2025 Mar 21;17(4):150. doi: 10.3390/toxins17040150 (PMC12030893; doi:10.3390/toxins17040150)
Supplement: Supplementary file 1 [file toxins-17-00150-s001.zip › toxins-3516253-supplementary.pdf]

toxins

MDPI

Supplementary Materials

Untargeted Metabolomic Analysis and Cytotoxicity of Extracts of the Marine Dinoflagellate *Amphidinium eilatiense* against Human Cancer Cell Lines

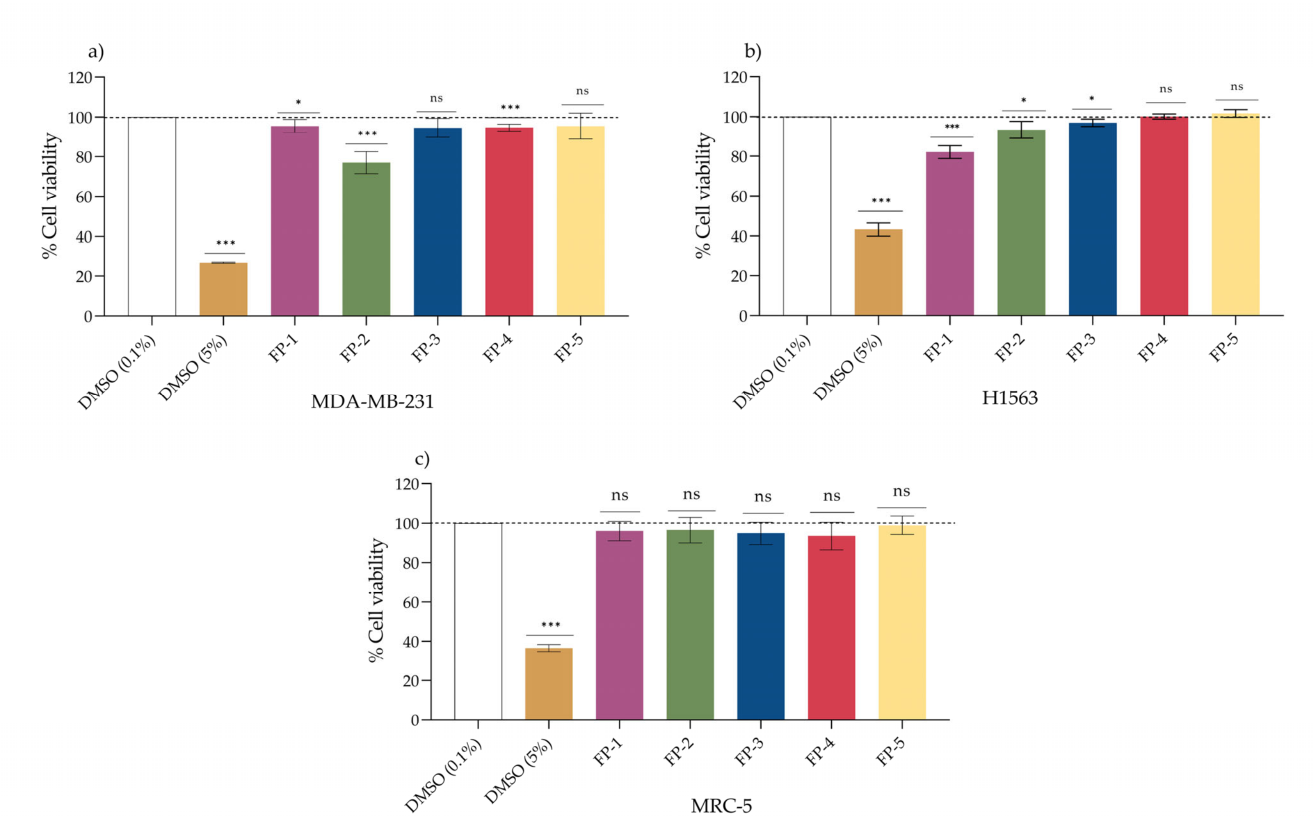

Figure S1. Comparison of the cytotoxic effect of polar fractions of *A. eilatiense* AeSQ181 on human cell lines. Treatment fraction concentration 50 µg mL^{-1} for exposure time 48 h. a) breast adenocarcinoma MDA-MB-231; b) lung adenocarcinoma H1563; c) human fibroblast normal cell line MRC-5. Negative control cells treated with 0.1% DMSO; positive control cells treated with 5% DMSO. Values are represented as mean ± SD; *n*=3. *p ≤ 0.05, ** ≤ 0.01,***p ≤ 0.001, and ns: no significant difference; * and ns indicate differences between fractions and negative control.

 https://doi.org/10.3390/xxxxx

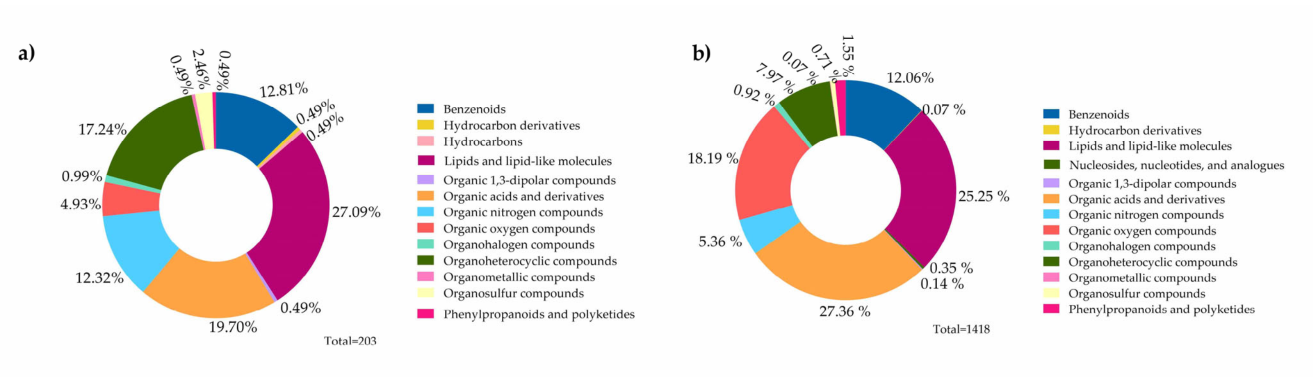

Figure S2. Pie chart showing the superclass of metabolites annotated from HRMS in positive ionization mode by untargeted metabolomic analysis applied to bioactive sub-fractions FNP-5 (a) and FNP-6 (b) from *A. eilatiense*.

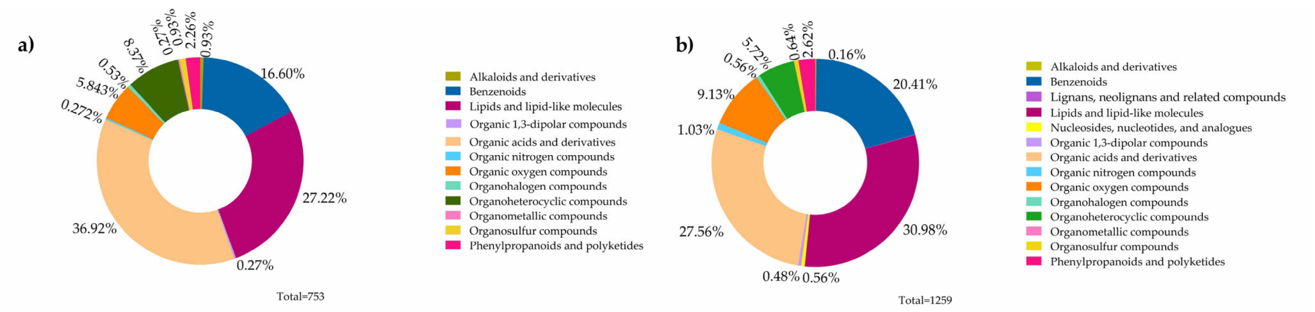

Figure S3. Pie chart showing the superclass of metabolites annotated from HRMS in negative ionization mode by untargeted metabolomic analysis applied to bioactive sub-fractions FNP-5 (a) and FNP-6 (b) from *A. eilatiense*.

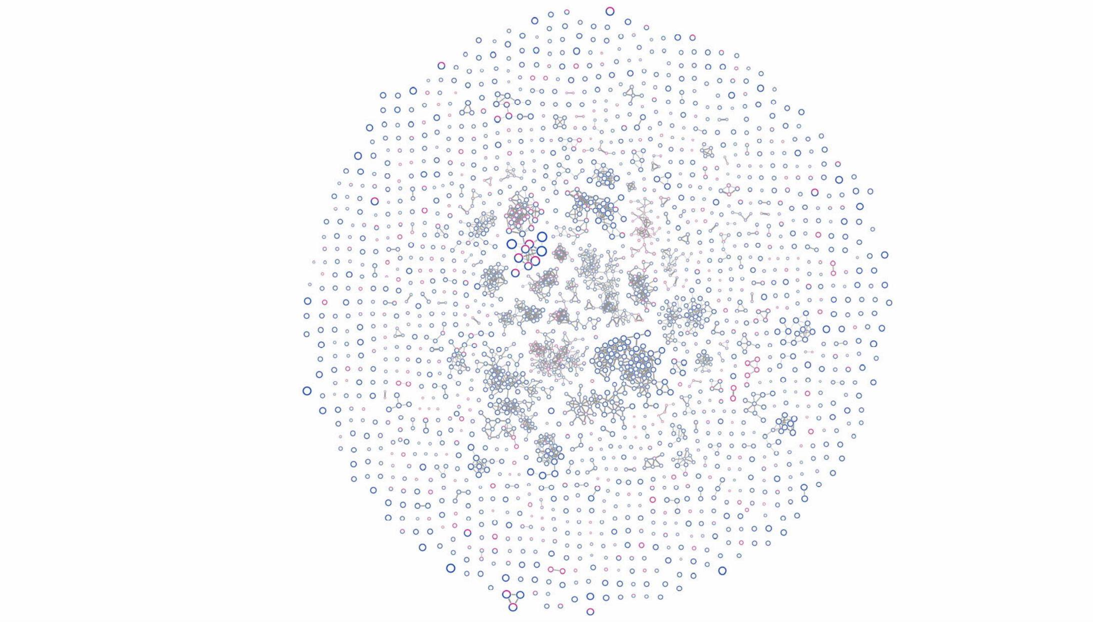

Figure S4. Molecular network showing abundance for each feature from subfractions FNP-5 (pink) and FNP-6 (blue) analyzed in positive ionization mode.

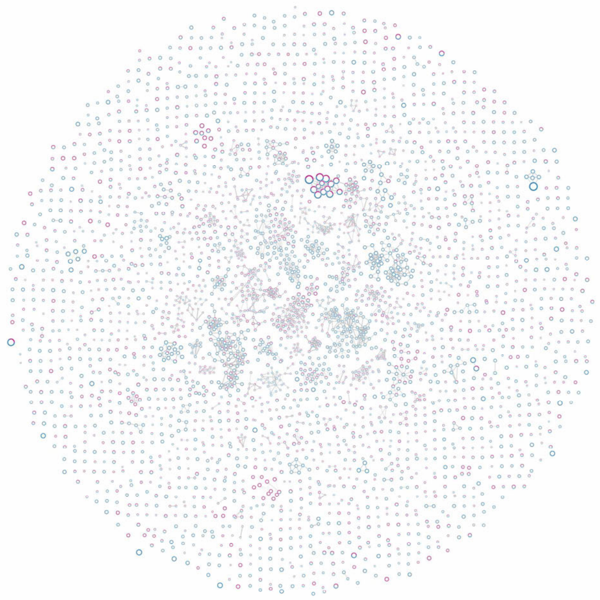

Figure S5. Molecular network shows abundance for each feature from subfractions FNP-5 (pink) and FNP-6 (blue) analyzed in negative ionization mode.

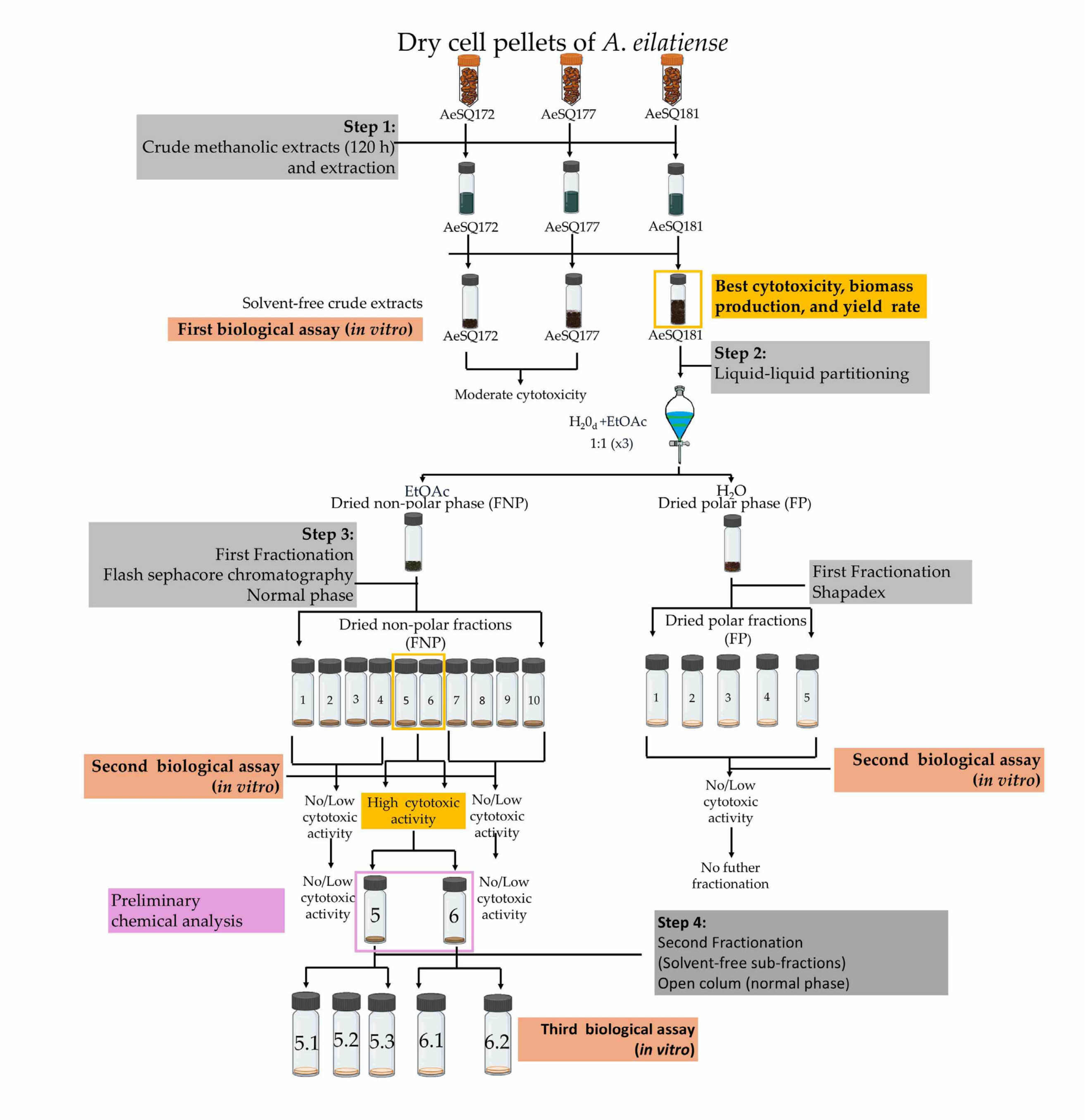

Figure S6. Flow diagram of extraction, partitioning, and fractionating of cell biomass of *A. eilatiense* AeSQ181 strain.

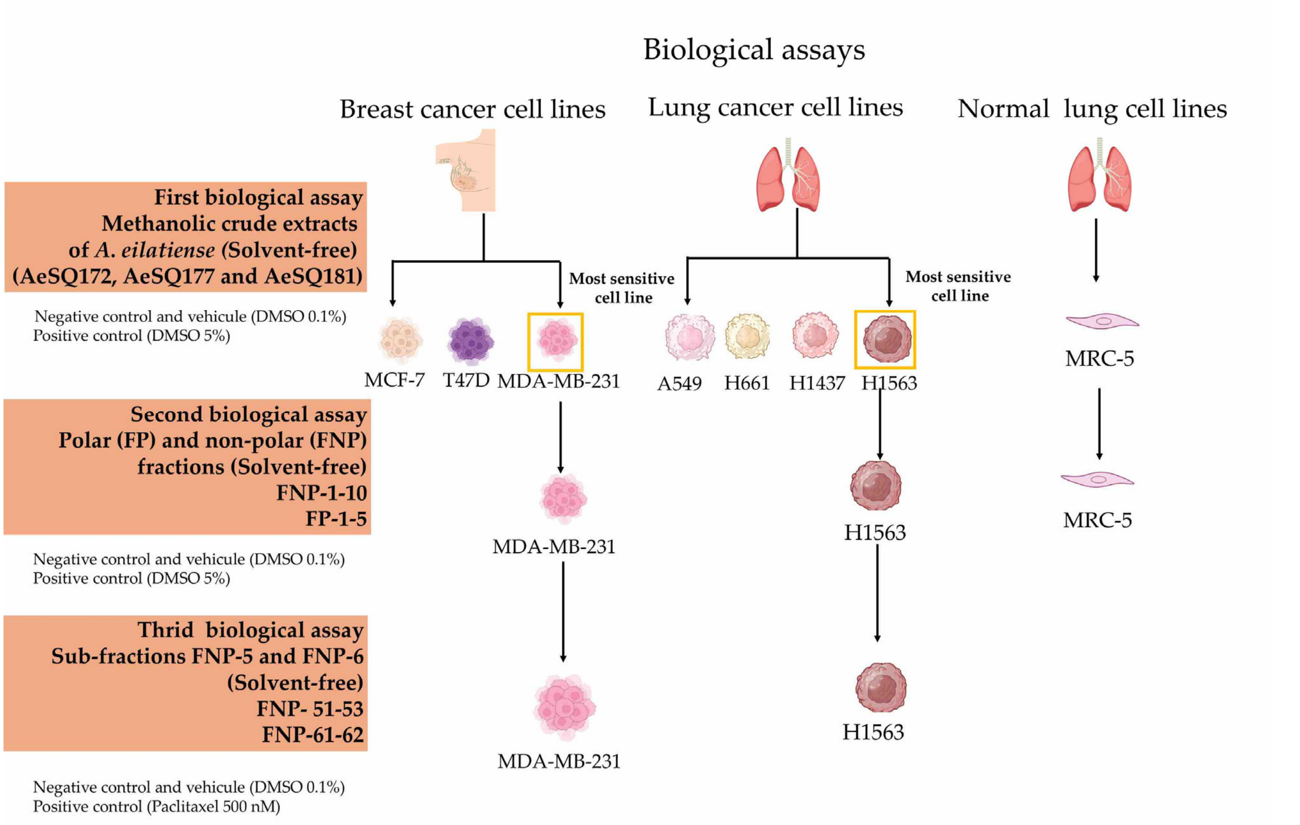

Figure S7. Workflow diagram for cell culture and bioassays of *A. eilatiense* AeSQ181 strains. The cell lines for cytotoxicity screening were selected based on their response to exposure to the extracts, fractions, and subfractions of *A. eilatiense* cells.

Detailed Description of the Untargeted Metabolomics Methodology Used for the Analysis of Active Fractions:

LC-MS2 vendor datasets with .d extension were converted to .mzXML format using the MSconvert tool from ProteoWizard, version 3.0.24033 (PMID: 23051804). Subsequently, the data were processed with MZmine software, version 3.9 (Schmid et al., 2023) for positive and negative ionization modes, separately.

The parameters for feature detection were as follows: mass detection (centroid, 2.0E5 and 2.0E3 for MS1 and MS2, respectively); ADAP chromatogram builder (minimum consecutive scans of 4, minimum intensity for consecutive scans of 2.0E5, minimum absolute height of 4.0E5, and *m*/*z* tolerance of 0.002 *m*/*z* or 5.0 ppm); chromatogram smoothing; ADAP chromatogram resolver (Myers et al., 2017) (S/N threshold of 10, minimum feature height of 2, coefficient/area threshold of 100, peak duration range: 0.01-0.5; and retention time (RT) wavelet range: 0.01-0.09); isotope peaks grouper (*m*/*z* tolerance set at 0.002 *m*/*z* or 5 ppm, retention time tolerance of 0.2 min, maximum charge of 1, and representative isotope set to most intense); RANSAC alignment (Pluskal et al., 2010) (*m*/*z* tolerance of 0.002 m/s or 5 ppm, retention time tolerance of 0.02 min, retention time tolerance after correction of 0.01 min, minimum number of points of 0.7 and threshold value of 0.2); gap filling. Three filters were applied: a) duplicate feature filter, b) removal of features detected in blank samples as well as in no-active fractions, and c) MS2-only filter to keep only the features with MS/MS spectra. The resulting feature list was exported as a feature quantification table (.csv) and MS2 spectral summary (.mgf) for downstream analyses. Feature-Based Molecular Networking (FBMN) (Nothias et al., 2020) was performed using the GNPS web platform. Preprocessed .csv and .mgf files generated by MZmine were

uploaded to GNPS for molecular networking and automated metabolite annotation via spectral matching. Key parameters included a precursor-ion and product-ion mass tolerance of 0.02 Da, a minimum cosine score of 0.6, and at least four shared fragment ions. DEREPLICATOR+ and MolDiscovery were applied within the GNPS platform, employing the following settings: precursor- and fragment-ion mass tolerances of 0.02 Da, a maximum charge of 1, and the complete database containing approximately 720,000 compounds. Additionally, a custom database derived from the Comprehensive Marine Natural Products Database (Lyu et al., 2021) was used for MolDiscovery analysis. Since MolDiscovery and DEREPLICATOR+ were incompatible with negative-mode datasets, MS-FINDER (Lai et al., 2018; Tsugawa et al., 2016) was employed instead. The MS2-containing .mgf file generated by MZmine was analyzed using the SIRIUS software GUI (version 5.8.5) (Dührkop et al., 2015) to determine the chemical formula and molecular structure (Dührkop et al., 2015).

Tentative metabolite annotations obtained through GNPS spectral library matching and *in silico* tools were automatically classified into chemical classes via the NPClassifier ontology (Kim et al., 2021). Outputs from FBMN, DEREPLICATOR+, CSI:FingerID, MolDiscovery, MS-FINDER, CANOPUS, and NPClassifier (Djoumbou Feunang et al., 2016) were integrated using a custom script based on MolNetEnhancer (Ernst et al., 2019) which is available here. The final output network was visualized in Cytoscape (version 3.10.1) (Shannon et al., 2003), with nodes colored at the SUPERCLASS level.

Some predicted metabolite annotations from *in silico* dereplication tools lacked precise chemical names and only provided SMILES strings. To address this, we used the SMILES-TO-IUPAC-name translator (STOUT) to convert SMILES strings into IUPAC names (Rajan et al., 2021).

Data analysis and visualization were primarily conducted using R software (version 4.3) and RStudio (version 2023.06.0). Detailed molecular information tables were created with the MetaboCoreUtils package (Rainer et al., 2022), and cheminf (Cao et al., 2008). The dplyr (Wickham et al., 2020) and data.table libraries (Barrett et al., 2024) served for data manipulation,. Pie charts were generated from the Plotly library (Inc, 2025).

References

1. Barrett, T., Dowle, M., Srinivasan, A., Gorecki, j., Chirico, M., T, H., & B, S. (2024). . *data.table: Extension of 'data.frame'. R package.* https://github.com/rdatatable/data.table.
2. Cao, Y., Charisi, A., Cheng, L.-C., Jiang, T., & Girke, T. (2008). ChemmineR: a compound mining framework for R. *Bioinformatics*, *24*(15), 1733-1734. https://doi.org/https://doi.org/10.1093/bioinformatics/btn307
3. Djoumbou Feunang, Y., Eisner, R., Knox, C., Chepelev, L., Hastings, J., Owen, G., Fahy, E., Steinbeck, C., Subramanian, S., & Bolton, E. (2016). ClassyFire: automated chemical classification with a comprehensive, computable taxonomy. *Journal of cheminformatics*, *8*, 1-20. https://doi.org/https://doi.org/10.1186/s13321-016-0174-y
4. Dührkop, K., Shen, H., Meusel, M., Rousu, J., & Böcker, S. (2015). Searching molecular structure databases with tandem mass spectra using CSI: FingerID. *Proceedings of the National Academy of Sciences*, *112*(41), 12580-12585. https://doi.org/https://doi.org/10.1073/pnas.1509788112
5. Ernst, M., Kang, K., Caraballo-Rodríguez, A., Nothias, L., Wandy, J., Chen, C., Wang, M., Rogers, S., Medema, M., & Dorrestein, P. (2019). MolNetEnhancer: enhanced molecular networks by integrating metabolome mining and annotation tools *Metabolites*, *9*, 144. https://doi.org/https://doi.org/10.3390/metabo9070144
6. Inc, P. T. (2025). *Collaborative data science Publisher: Plotly Technologies Inc*. https://plot.ly
7. Kim, H. W., Wang, M., Leber, C. A., Nothias, L.-F., Reher, R., Kang, K. B., Van Der Hooft, J. J., Dorrestein, P. C., Gerwick, W. H., & Cottrell, G. W. (2021). NPClassifier: a deep neural network-based structural classification tool for natural products. *Journal of Natural Products*, *84*(11), 2795-2807. https://doi.org/https://doi.org/10.1021/acs.jnatprod.1c00399

8. Lai, Z., Tsugawa, H., Wohlgemuth, G., Mehta, S., Mueller, M., Zheng, Y., Ogiwara, A., Meissen, J., Showalter, M., & Takeuchi, K. (2018). Identifying metabolites by integrating metabolome databases with mass spectrometry cheminformatics. *Nature methods*, *15*(1), 53-56. https://doi.org/https://doi.org/10.1038/nmeth.4512
9. Lyu, C., Chen, T., Qiang, B., Liu, N., Wang, H., Zhang, L., & Liu, Z. (2021). CMNPD: a comprehensive marine natural products database towards facilitating drug discovery from the ocean. *Nucleic Acids Research*, *49*(D1), D509-D515. https://doi.org/https://doi.org/10.1093/nar/gkaa763
10. Myers, O. D., Sumner, S. J., Li, S., Barnes, S., & Du, X. (2017). One step forward for reducing false positive and false negative compound identifications from mass spectrometry metabolomics data: new algorithms for constructing extracted ion chromatograms and detecting chromatographic peaks. *Analytical chemistry*, *89*(17), 8696-8703. https://doi.org/https://doi.org/10.1021/acs.analchem.7b00947
11. Nothias, L.-F., Petras, D., Schmid, R., Dührkop, K., Rainer, J., Sarvepalli, A., Protsyuk, I., Ernst, M., Tsugawa, H., & Fleischauer, M. (2020). Feature-based molecular networking in the GNPS analysis environment. *Nature methods*, *17*(9), 905-908. https://doi.org/https://doi.org/10.1038/s41592-020-0933-6
12. Pluskal, T., Castillo, S., Villar-Briones, A., & Orešič, M. (2010). MZmine 2: modular framework for processing, visualizing, and analyzing mass spectrometry-based molecular profile data. *BMC bioinformatics*, *11*, 1-11. https://doi.org/https://doi.org/10.1186/1471-2105-11-395
13. Rainer, J., Vicini, A., Salzer, L., Stanstrup, J., Badia, J. M., Neumann, S., Stravs, M. A., Verri Hernandes, V., Gatto, L., & Gibb, S. (2022). A modular and expandable ecosystem for metabolomics data annotation in R. *Metabolites*, *12*(2), 173. https://doi.org/https://doi.org/10.3390/metabo12020173
14. Rajan, K., Zielesny, A., & Steinbeck, C. (2021). STOUT: SMILES to IUPAC names using neural machine translation. *Journal of cheminformatics*, *13*(1), 34. https://doi.org/https://doi.org/10.1186/s13321-021-00512-4
15. Schmid, R., Heuckeroth, S., Korf, A., Smirnov, A., Myers, O., Dyrlund, T. S., Bushuiev, R., Murray, K. J., Hoffmann, N., & Lu, M. (2023). Integrative analysis of multimodal mass spectrometry data in MZmine 3. *Nature biotechnology*, *41*(4), 447-449. https://doi.org/https://doi.org/10.1038/s41587-023-01690-2
16. Shannon, P., Markiel, A., Ozier, O., Baliga, N. S., Wang, J. T., Ramage, D., Amin, N., Schwikowski, B., & Ideker, T. (2003). Cytoscape: a software environment for integrated models of biomolecular interaction networks. *Genome research*, *13*(11), 2498-2504. https://doi.org/https://doi.org/10.1101/gr.1239303
17. Tsugawa, H., Kind, T., Nakabayashi, R., Yukihira, D., Tanaka, W., Cajka, T., Saito, K., Fiehn, O., & Arita, M. (2016). Hydrogen rearrangement rules: computational MS/MS fragmentation and structure elucidation using MS-FINDER software. *Analytical Chemistry* *88*(16), 7946-7958. https://doi.org/https://doi.org/10.1021/acs.analchem.6b00770
18. Wickham, H., François, R., Henry, L., Müller, K., & Vaughan, D. (2020). Dplyr: A grammar of data manipulation.
